# Acid–base physiology over tidal periods in the mussel *Mytilus edulis*: size and temperature are more influential than seawater pH

**DOI:** 10.1098/rspb.2018.2863

**Published:** 2019-02-20

**Authors:** Stephanie Mangan, Rod W. Wilson, Helen S. Findlay, Ceri Lewis

**Affiliations:** 1 Biosciences, University of Exeter, Geoffrey Pope Building, Stocker Road, Exeter EX4 4QD, UK; 2 Plymouth Marine Laboratory, Prospect Place, West Hoe, Plymouth PL1 3DH, UK

**Keywords:** ocean acidification, acid–base balance, emersion, multi-stressor

## Abstract

Ocean acidification (OA) studies to date have typically used stable open-ocean pH and CO_2_ values to predict the physiological responses of intertidal species to future climate scenarios, with few studies accounting for natural fluctuations of abiotic conditions or the alternating periods of emersion and immersion routinely experienced during tidal cycles. Here, we determine seawater carbonate chemistry and the corresponding *in situ* haemolymph acid–base responses over real time for two populations of mussel (*Mytilus edulis*) during tidal cycles, demonstrating that intertidal mussels experience daily acidosis during emersion. Using these field data to parameterize experimental work we demonstrate that air temperature and mussel size strongly influence this acidosis, with larger mussels at higher temperatures experiencing greater acidosis. There was a small interactive effect of prior immersion in OA conditions (pH_NBS_ 7.7/pCO_2_ 930 µatm) such that the haemolymph pH measured at the start of emersion was lower in large mussels exposed to OA. Critically, the acidosis induced in mussels during emersion *in situ* was greater (ΔpH approximately 0.8 units) than that induced by experimental OA (ΔpH approximately 0.1 units). Understanding how environmental fluctuations influence physiology under current scenarios is critical to our ability to predict the responses of key marine biota to future environmental changes.

## 1. Introduction

In an era of unprecedented global ocean and climatic change, there is growing interest in understanding the physiological mechanisms by which environmental conditions influence species' performance and how that varies over space and time. Coastal systems are characterized by natural fluctuations in abiotic conditions occurring on a daily (tidal), seasonal, and annual basis [1,2]. This is particularly evident within temperate regions where strong seasonal stratification in addition to upwelling events, temperature, salinity, photosynthesis, respiration, and tidal cycles can result in fluctuations of seawater pH [3,4]. This variability is predicted to intensify as atmospheric CO_2_ rises, with ocean acidification (OA), warming, and other climatic changes expected to exacerbate intertidal extremes [5]. For intertidal organisms these fluctuations are coupled with alternating periods of emersion (out of water) and immersion (back in water) which collectively have the potential to act synergistically and pose significant physiological stress [6], with potential knock-on effects for population distribution, growth rates, and fitness [7]. Understanding how an organism responds to variability in natural environmental conditions is therefore fundamental for informing predictions of future vulnerabilities.

There is now a wealth of evidence suggesting that OA and warming have the potential to negatively impact physiology, growth, and reproduction across a wide range of marine fauna [8,9]. However, for intertidal organisms tidal oscillations influence physiological and behavioural changes such as metabolic rate [10], heart rate [11], valve opening [12], and intermediary metabolite cycles [13] with the emersion phase of their habitat having the potential to pose the greatest physiological stress. During immersion mussels have access to food, dissolved oxygen, and their body temperature remains relatively stable, following that of the surrounding seawater. However, during periods of emersion, they are unable to feed, have limited access to oxygen owing to the closure of valves to prevent desiccation, and body temperature is dependent on air temperature, the effects of solar irradiance, evaporation, and wind speed [14], with body size also playing a role [15]. In addition, a reduction of internal oxygen levels in closed bivalves has been shown to shift metabolism to anaerobic pathways, which in turn with the accumulation of execratory metabolic products can lead to significant hypercapnia and acid–base disturbances [7,16]. The ability to compensate these acid–base disturbances and maintain cellular homeostasis has been suggested to play an important role in the future survival and distribution of a given species [17].

In coastal areas, recent observations have demonstrated that pH can fluctuate by 1.0 pH unit [18], far exceeding the global average predictions for the end of the century due to OA [19]. With seawater pH predicted to decrease by a further 0.3–0.4 units by the end of the century [20] and fluctuations in carbonate parameters expected to intensify [5], OA may pose significant additional physiological stress on intertidal invertebrates. The influence of OA on the acid–base physiology of marine fauna is well documented, mostly following prolonged exposures to stable future open-ocean pH values [21]. For example, in the mussel *Mytilus edulis* a CO_2_-induced decrease in seawater pH induces an extracellular acidosis with no compensatory increase in bicarbonate ions $\left(\mathrm{HC}O_{3}^{-}\right)$ [22–24]. This acidosis during immersion can then exacerbate hypercapnia during emersion periods for intertidal organisms, as recently demonstrated in laboratory exposures for the Sydney rock oyster *Saccostrea glomerata* [7]. Extracellular acidosis may also be induced via temperature changes during emersion [25]. Globally, average surface temperatures have increased by 0.7°C and are projected to rise by up to 4°C by the end of the century [20] potentially posing a significant threat to intertidal organisms exposed for substantial periods of the day. Body temperature within aerially exposed mussels has been shown to fluctuate by 20°C within 12 h [26], which can also affect the kinetics of metabolism and hence also lead to metabolic acidosis [16].

While combined effects of temperature and OA on the physiology of intertidal organisms have been demonstrated [27,28], few studies have incorporated field monitoring of acid–base status over a tidal cycle to determine the conditions organisms currently experience. Here, we follow acid–base disturbances *in situ* in real time over tidal cycles for two populations of *M. edulis* and then use these field data to inform laboratory studies to test the hypotheses that; (i) prior exposure to OA conditions will increase acid–base disturbances during subsequent emersion and (ii) acid–base disturbances during emersion will be greater at higher temperatures and with larger body size. This will aid our understanding of the present variability experienced by coastal organisms and help to inform predictions of future responses to an additional climate signal.

## 2. Material and methods

### (a) Field monitoring

Monitoring took place at Starcross (SC), South Devon, a sheltered muddy estuary (50°37′03″ N −3°26′56″ W) and Port Gaverne (PG), North Cornwall, an exposed rocky shore (50°35′38″ N −4°49′26″ W) in November 2014 (Starcross only), April, July, and September 2015. Both sites contain large intertidal populations of *M. edulis* experiencing semidiurnal tidal cycles. Sampling took place every 30 min over a 12 h daytime period to include a tidal cycle of one high and one low tide. To differentiate between population differences and environmental influence, during the July sample at PG, additional mussels from SC were transposed the previous day in a mesh bag and sampled alongside the PG mussels.

At both sites seawater was collected every 30 min during periods of immersion for assessment of pH_NBS_, temperature, salinity, and dissolved inorganic carbon (DIC). Seawater pH_NBS_ was measured to ±0.01 pH units immediately on collection using a Metrohm (826 pH mobile) pH electrode calibrated at the appropriate temperature prior to use with NBS buffers. Salinity and temperature were measured at an accuracy of ±0.1 psu and ±0.1°C using a Mettler Toledo SG7 SevenGo pro conductivity meter. Temperature was additionally monitored to an accuracy of ±0.1°C using HOBO Pendant^®^ Temperature/Light Data Loggers for the July and September samples at both sites. DIC samples were preserved on site with saturated HgCl_2_ [29] and analysed for DIC using a custom-built system following Lewis *et al*. [30]. Data outputs were analysed using Logger Pro version 3.2 software with a measurement precision of ±3 µM. Total alkalinity and *p*CO_2_ were calculated using CO2sys [31] using K1 and K2 values refitted by Dickson & Millero [32] from Mehrbach [33], and KSO_4_ determined by Dickson [34].

For the mussel acid–base measurements, haemolymph from the posterior abductor muscle was extracted from three mussels every 30 min, with each mussel sampled just once. Following immediate measurements of haemolymph pH (Metrohm 826 pH mobile and electrode, as above) was transferred to glass micro-hematocrit capillary tubes and placed on ice before being taken back to the laboratory for analysis of total CO_2_ using a Corning 965 CO_2_ analyser (Corning Ltd., UK) calibrated with a 10 mM NaHCO_3_ solution (with an accuracy of 0.1 mM). Acid–base parameters were then calculated using a modified version of the Henderson–Hasselbalch equation using the first dissociation constant (pK) for carbonic acid and solubility constant (αCO_2_) for CO_2_ derived from Truchot [35].

### (b) Laboratory experiments

Two size classes of adult *M. edulis* (37–50 mm and 60–79 mm shell length) were collected from the mid-shore of the intertidal region in Starcross. Barnacles were carefully removed, then mussels were held in a recirculating system of artificial seawater filtered to 1 µm (©Tropic marine), (pH_NBS_ 8.10, pCO_2_ 340 µatm, salinity 32, at 15 ± 0.5°C, photoperiod 12.12) for a minimum of 7 days before experimentation and fed 5000 cells ml^−1^ of dried *Isochrysis* daily. To reach the target pH_NBS_ of 7.70 (pCO_2_ 930 µatm) a computerized control system (Aqua Medic, Germany) was used to control seawater pH, whereby gaseous CO_2_ was fed into the seawater via a solenoid valve until pH reached the target level. Seawater pH_NBS_ was independently monitored with a Metrohm 826 pH_NBS_ mobile electrode and meter. All seawater was aerated at a low constant rate to maintain normoxic oxygen levels but without interfering with the control of seawater CO_2_ and pH. Seawater samples were collected for assessment of pH_NBS_ and DIC as described above.

The two size classes comprising small (37–50 mm, *N* = 78) and large (60–79 mm, *N* = 78) mussels, were randomly divided in half, and each half was exposed to seawater at either pH_NBS_ 8.10 or pH_NBS_ 7.70 for one week at 13°C. To remove any influence of repeat sampling on physiological parameters, mussels were only ever sampled once and then discarded. Mussels were subsequently removed from the water (for the emersion period) in a temperature controlled room at either 7°C, 13°C, 20°C, or 28°C (±0.5°C) for 6 h. Following emersion, the remaining un-sampled mussels were then placed back into their respective treatment seawater to investigate recovery over the following 6 h. Every 45 min during emersion (three mussels per time point) and every hour during recovery (four mussels per time point) haemolymph was extracted for assessment of acid–base response as described above.

### (c) Statistical analysis

For field data, correlations between haemolymph parameters and physico-chemical data were investigated using regression analysis in Minitab v18. For the laboratory experiment, haemolymph measurements were quality checked using standard methods (see electronic supplementary material, Appendix text) and data were tested for normality using the Shapiro–Wilk test and found to be non-normal. No transformations were able to normalize the data, hence PERMANOVA tests were performed, which make no assumption about distribution. In order to evaluate dispersion across groups, we used PERMDISP function, a distance-based test for homogeneity of multivariate dispersions, using deviations from the median. The results of these tests are reported in electronic supplementary material, tables S4–S9 before each PERMANOVA result, but in brief there were no significant differences in dispersions across groups (*p* > 0.05), except for the recovery pCO_2_ data (*F* = 1.8507, *p* = 0.029). Euclidean distance was used to create a resemblance matrix for each of the acid–base parameters before PERMANOVA was performed, testing for effects of mussel size, experimental time, temperature treatment, and pH treatment. Size, time, temperature, and pH were used as factors, the data were analysed as multivariate and each mussel result was treated as an individual ‘sample’ in the analysis. Analysis was conducted in Primer 6 with PERMANOVA.

## 3. Results

### (a) Field monitoring

Over the daily tidal cycles, seawater conditions were only measured during the period while the mussel bed was submerged. Across all sites and seasons, the daily range of conditions (max – min) over each of these measured periods was less than 13 Δ°C, less than 10 Δpsu, less than 0.5 ΔpH_NBS_, and less than 611 Δµatm (summarized in table 1). There were no clear daily patterns in the data except for temperature, which increased slightly during the daytime, as would be expected due to solar heating, and there were no differences in daily conditions between the two sites (see electronic supplementary material, figures S1 and S2). Our data recorded the highest sea surface temperatures (SST) in July (21 ± 0.7°C at PG and 19 ± 0.4°C at SC; electronic supplementary material, figures S1 and S2), and the coldest in November (12 ± 0.1°C; see electronic supplementary material, figures S1 and S2) at SC (no November measurement was available for PG). At PG our data recorded the lowest salinity in July (23.4 ± 0.4 psu; electronic supplementary material, figure S2), but at SC the lowest salinity was in November (26.2 ± 0.6 psu; electronic supplementary material, figure S1). Throughout the year seawater pH_NBS_ showed no clear seasonal pattern except for a decrease during July at PG which coincided with the low salinity measurement (table 1 and electronic supplementary material, figure S2). On average, the calculated mean seawater pCO_2_ (table 1, also see electronic supplementary material, figures S1 and S2 and supplementary dataset S1) was lower than mean atmospheric CO_2_ values (approx. 400 µatm as measured at Penlee Point Atmospheric Observatory, http://www.westernchannelobservatory.org.uk/penlee/). However pCO_2_ levels up to approximately 650 µatm were calculated at SC, when pH measurements were below pH_NBS_ 8.0. There was similar variability at both sites throughout the year in terms of pH and pCO_2_.

**Table 1.** Seawater conditions at the two sites on each of the measurement days: mean ± s.d. (minimum – maximum).

site	Port Gaverne			Starcross			
date	15 Apr	15 July	15 Sep	14 Nov	15 Apr	15 July	15 Sep
temp. (°C)	15.6 ± 3.2 (11.1–21.6)	21.9 ± 2.1 (18.4–26.2)	15.4 ± 0.4 (14.7–16.1)	11.7 ± 0.6 (10.7–12.6)	13.6 ± 3.7 (6.0–18.5)	19.1 ± 1.4 (16.1–22.0)	17.9 ± 2.0 (13.7–20.1)
pH_NBS_	8.17 ± 0.05 (8.10–8.24)	8.14 ± 0.06 (8.01–8.22)	8.15 ± 0.07 (7.97–8.28)	8.15 ± 0.14 (7.90–8.29)	8.17 ± 0.08 (8.07–8.36)	8.16 ± 0.07 (8.08–8.34)	8.09 ± 0.12 (7.86–8.35)
salinity	29.8 ± 2.3 (25.3–32.6)	23.3 ± 1.6 (20.7–24.8)	27.7 ± 2.8 (22.1–31.2)	26.2 ± 2.4 (22.8–29.6)	30.4 ± 2.0 (27.3–33.0)	28.9 ± 2.6 (23.6–33.0)	31.0 ± 2.4 (24.9–32.9)
TCO_2_ (µmol kg^−^¹)	2031 ± 30 (1969–2069)	2019 ± 45 (1967–2096)	2087 ± 34 (2055–2146)	1932 ± 108 (1754–2093)	2006 ± 75 (1877–2108)	2071 ± 116 (1876–2236)	1998 ± 157 (1610–2157)
TA (µmol kg^−^¹)	2286 ± 38 (2228–2342)	2255 ± 60 (2170–2373)	2321 ± 51 (2216–2413)	2123 ± 154 (1844–2306)	2250 ± 86 (2123–2387)	2349 ± 112 (2116–2497)	2245 ± 148 (1984–2434)
pCO_2_ (µatm)	292 ± 33 (243–334)	346 ± 53 (276–452)	321 ± 58 (238–482)	307 ± 109 (195–534)	290 ± 55 (166–366)	318 ± 63 (183–398)	364 ± 128 (142–647)

### (b) Mussel acid–base physiology during *in situ* emersion

**Figure 1.**
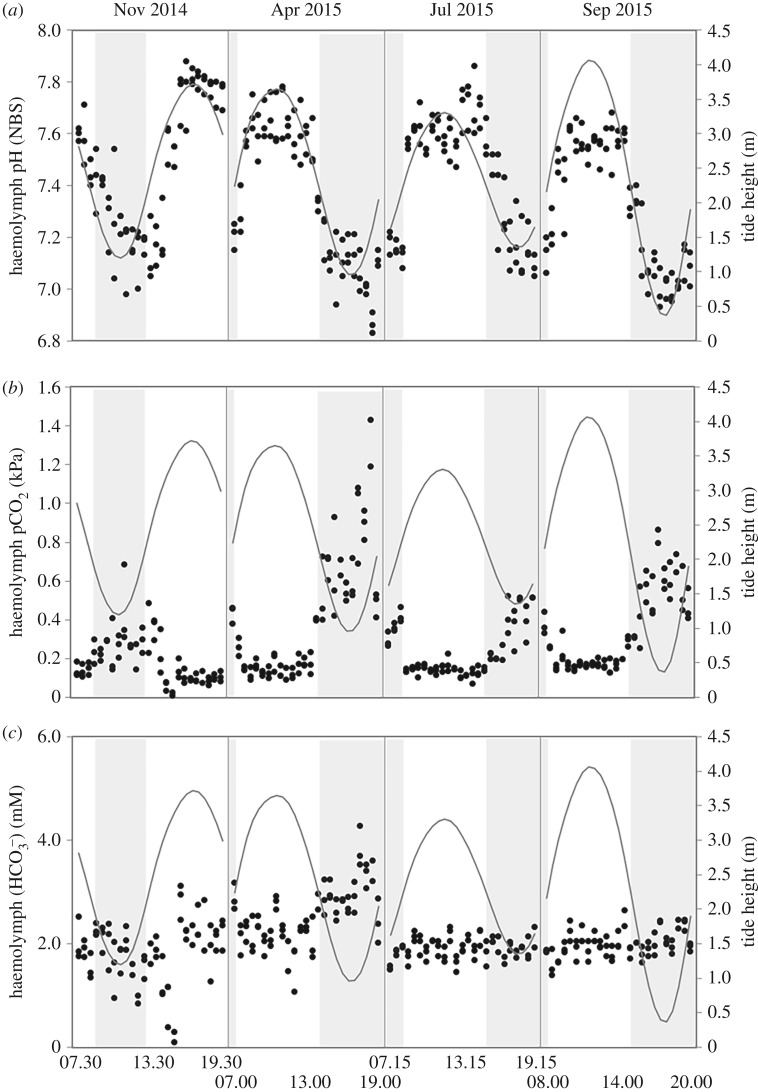
Acid–base parameters of mussels (black dots) from Starcross during the sampled tidal cycles (grey line = tide height) in November 2014, April 2015, July 2015, and September 2015 showing: (*a*) haemolymph pH_NBS_; (*b*) haemolymph pCO_2_ (KPa); and (*c*) haemolymph $\mathrm{HC}O_{3}^{-}$ concentration (mM). Shaded sections represent periods of emersion.

**Figure 2.**
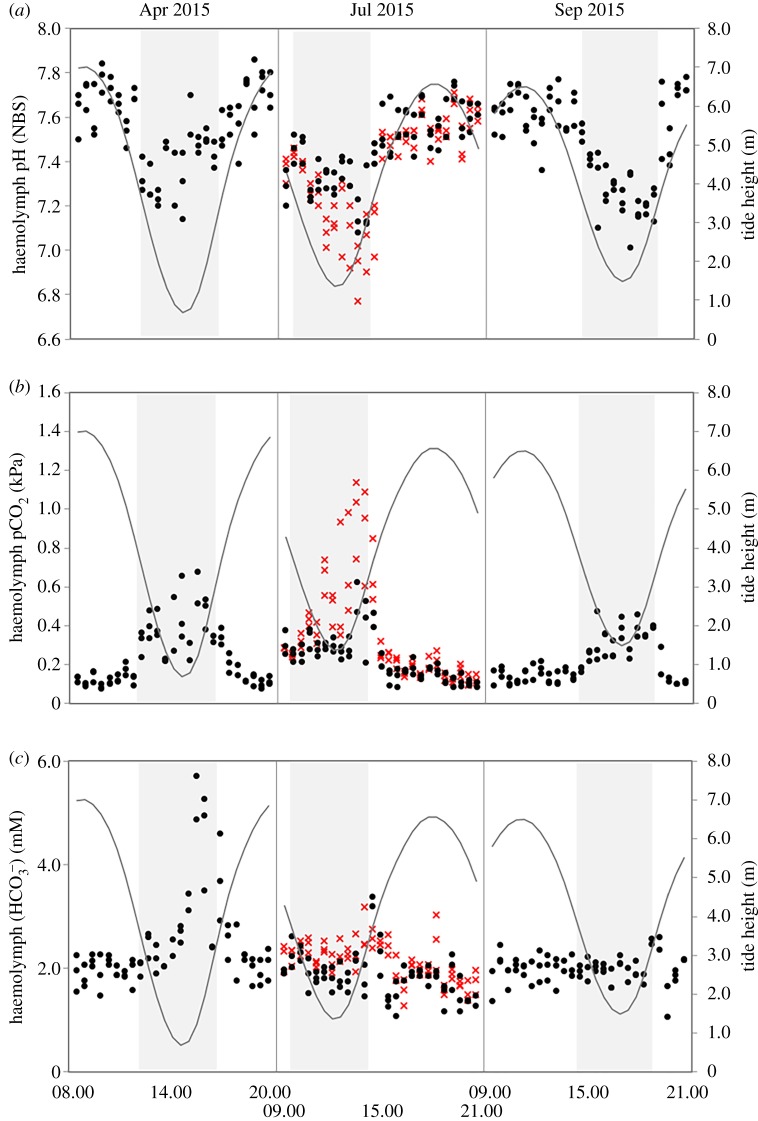
Acid–base parameters of mussels (black dots) from Port Gaverne during the sampled tidal cycles (grey line = tide height) in April 2015, July 2015, and September 2015 showing: (*a*) haemolymph pH_NBS_; (*b*) haemolymph pCO_2_ (KPa); and (*c*) haemolymph $\mathrm{HC}O_{3}^{-}$ concentration (mM). Shaded sections represent periods of emersion. Red crosses represent the acid–base parameters for mussels transplanted from Starcross during the July sampling. (Online version in colour.)

During aerial exposure (low tide), haemolymph pH_NBS_ decreased significantly at both sites (*p* < 0.001, summarized in electronic supplementary material, table S1), although the magnitude of decrease was greater in mussels from SC (figure 1) compared to PG (figure 2), such that at SC (larger mussels with shell length range of 52.6–78.4 mm), haemolymph pH_NBS_ decreased from a maximum pH_NBS_ 7.58 to a minimum pH_NBS_ 6.87, while at PG (smaller mussels with shell length range of 30.7–47.1 mm), haemolymph pH_NBS_ decreased from a maximum pH_NBS_ 7.53 to a minimum pH_NBS_ 7.15. This corresponded with an increase in haemolymph pCO_2_ (figures 1 and 2), while haemolymph $\mathrm{HC}O_{3}^{-}$ concentration remained relatively stable over a tidal cycle except during April when it increased during emersion (figures 1 and 2). In April, at both sites, there was an increase in haemolymph $\mathrm{HC}O_{3}^{-}$ during emersion, reaching a peak just before mussels were re-submerged by the tide. This increase in haemolymph $\mathrm{HC}O_{3}^{-}$ was most evident at PG (figure 2) reaching a maximum of 6.2 mM, but also occurred at SC (reaching a maximum of 4.3 mM, figure 1). For the SC mussels this April haemolymph $\mathrm{HC}O_{3}^{-}$ peak coincided with a particularly high haemolymph pCO_2_ value of 1.43 kPa, more than double the highest haemolymph pCO_2_ value recorded for any of the other sampling time points. There was no evidence of a haemolymph $\mathrm{HC}O_{3}^{-}$ increase during emersion periods in any of the other months studied.

The larger SC mussels transposed to PG for the July sampling exhibited a more pronounced extracellular acidosis during emersion than the PG population: SC minimum pH_NBS_ 6.91 compared to the PG minimum pH_NBS_ of 7.15 (figure 2). This was coupled with increased haemolymph pCO_2_ levels, but with stable haemolymph $\mathrm{HC}O_{3}^{-}$ concentrations (figure 2). These differences in response between populations is statistically significant between PG and SC (see electronic supplementary material, Appendix text and electronic supplementary material, tables S1–S3).

### (c) Mussel acid–base physiology during *in situ* immersion

When submerged by the tide, mussel haemolymph pH_NBS,_ pCO_2_, and $\mathrm{HC}O_{3}^{-}$ were all found to have significant correlations with seawater pH at SC (haemolymph pH_NBS_: *r*^2^ = 0.43, d.f. = 163, *p* < 0.0001; haemolymph pCO_2_: *r*^2^ = 0.28, d.f. = 155, *p* < 0.0001; haemolymph $\mathrm{HC}O_{3}^{-}$: *r*^2^ = 0.036, d.f. = 155, *p* = 0.017) but not at PG (haemolymph pH_NBS_: *r*^2^ = 0.03, d.f. = 131, *p* = 0.538; haemolymph pCO_2_: *r*^2^ = 0.002, d.f. = 124, *p* = 0.628; haemolymph $\mathrm{HC}O_{3}^{-}$: *r*^2^ = 0.023, d.f. = 125, *p* = 0.089). At PG, haemolymph pH_NBS_ and pCO_2_ were most significantly correlated (albeit a weak correlation) with temperature—both seawater temperature (haemolymph pH_NBS_: *r*^2^ = 0.174, d.f. = 131, *p* < 0.0001; haemolymph pCO_2_: *r*^2^ = 0.22, d.f. = 124, *p* < 0.0001) and haemolymph temperature (haemolymph pH_NBS_: *r*^2^ = 0.19, d.f. = 131, *p* < 0.0001; haemolymph pCO_2_: *r*^2^ = 0.25, d.f. = 124, *p* < 0.0001), which were themselves highly correlated (*r*^2^ = 0.94, d.f. = 133, *p* < 0.0001). Haemolymph $\mathrm{HC}O_{3}^{-}$ at PG did not correlate significantly with any other environmental factor measured, remaining relatively stable during periods of immersion.

Both populations showed similar acid–base status during periods of immersion in seawater (figure 2), as demonstrated when mussels from SC were transposed to PG during the July sampling (*t*-test comparing haemolymph acid–base parameters of the two populations at high tide point: pH *T* = −1.5509, d.f. = 87, *p* = 0.1245; pCO_2_ *T* = 1.83235, d.f. = 88, *p* = 0.0703; $\mathrm{HC}O_{3}^{-}$ *T* = 1.97844, d.f. = 87, *p* = 0.0510).

### (d) Laboratory manipulation experiments

**Figure 3.**
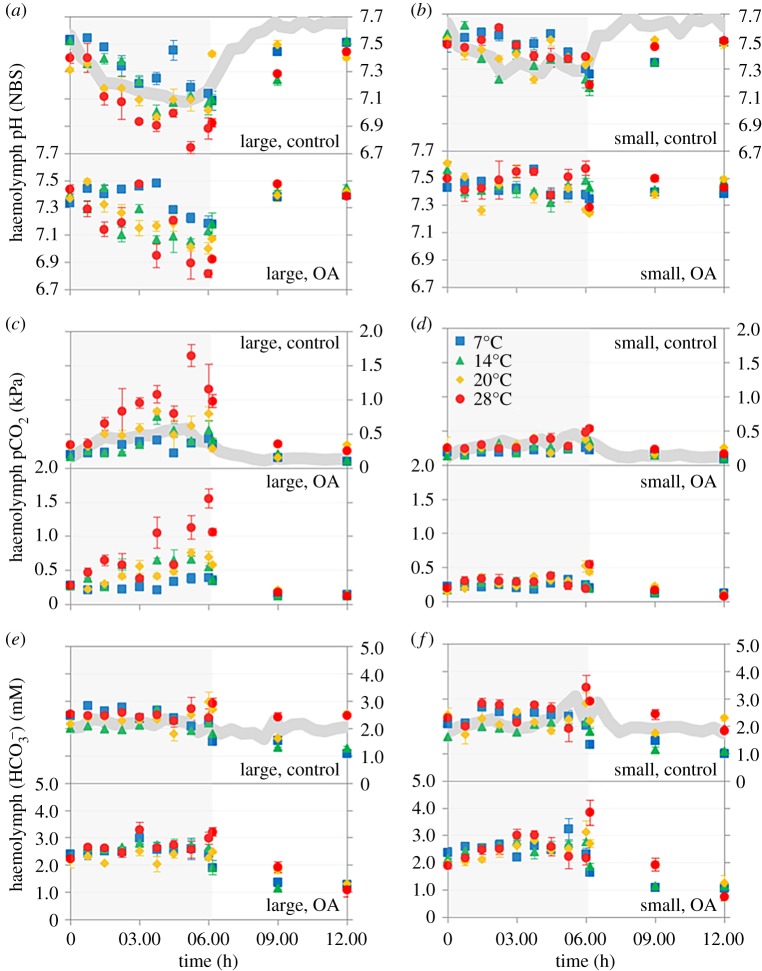
Acid–base parameters in two size classes of mussels: large (*a*,*c*,*e*) and small (*b*,*d*,*f*) during emersion and recovery experiments at different temperatures: 7°C (blue squares), 14°C (green triangles), 20°C (yellow diamonds), and 28°C (red circles), and with different pre-exposure to pH conditions: ‘Control’ pH_NBS_ 8.1 (top panels) and ‘OA’ pH_NBS_ 7.7 (bottom panels). Error bars represent standard error. Shaded sections represent periods of emersion. Also shown as thick grey lines, thickness represents 1 s.d. around the mean for each acid–base parameter from all field data, from large mussels collected at Starcross (*a*,*c*,*e*) and small mussels collected at Port Gaverne (*b*,*d*,*f*).

Seawater carbonate chemistry data for the laboratory experiments is summarized in the electronic supplementary material, Appendix text, and electronic supplementary material, table S10. Prior exposure to simulated OA conditions for one week had no significant effect on haemolymph pH during emersion when considered as a single factor (OA: Pseudo-*F*_(1,995)_ = 0.082, *p* = 0.786). However, there was a significant interaction with size and emersion time for haemolymph pH_NBS_ (OA × size × time: Pseudo-*F*_(8,999)_ = 2.683, *p* = 0.009) such that at the first time point, haemolymph pH_NBS_ was lower in mussels that had been exposed to OA conditions compared to the controls; this was only significant in the large mussels (control T0 pH_NBS_ = 7.48 ± 0.06 (mean ± s.d.), OA T0 pH_NBS_ = 7.38 ± 0.04 (mean ± s.d.)). Haemolymph pH_NBS_ then decreased over the 6 h emersion period, with the largest decrease again occurring in the large mussels (figure 3). There was much less of a decrease in haemolymph pH_NBS_ in the smaller mussels (from both control and OA treatments), and no evidence in either size class of the prior exposure to OA conditions by the end of the emersion period. Haemolymph pCO_2_ was also not significantly affected by the prior OA treatment (OA: Pseudo-*F*_(1,999)_ = 1.182, *p* = 0.283) or its interaction with size and emersion time (OA × size × time = Pseudo-*F*_(8,999)_ = 1.561, *p* = 0.158), with haemolymph pCO_2_ increasing in the large mussels through time, but not in response to OA, and showing only a small increase through time in the small mussels. Haemolymph $\mathrm{HC}O_{3}^{-}$ was significantly impacted by the OA treatment (OA: Pseudo-*F*_(1,995)_ = 60.019, *p* = 0.001), such that it was on average higher during emersion in mussels that had been previously exposed to OA conditions compared to the controls. However, there was no significant interaction between OA, size, and emersion time (OA × size × time: Pseudo-*F*_(8,999)_ = 0.465, *p* = 0.888), with haemolymph $\mathrm{HC}O_{3}^{-}$ remaining relatively constant through the emersion period and at similar levels in both small and large mussels (figure 3). Full statistical results are provided in the electronic supplementary material text and electronic supplementary material, tables S4–S9.

Air temperature strongly affected all acid–base parameters during the 6 h emersion period. Haemolymph pH_NBS_ decreased and pCO_2_ increased strongly as temperature increased, with this being most evident in the larger mussels (Temp effect on haemolymph pH: Pseudo-*F*_(3,999)_ = 64.686, *p* = 0.001; Temp × size × time for haemolymph pH: Pseudo-*F*_(24,998)_ = 2.942, *p* = 0.001; Temp effect on haemolymph pCO_2_: Pseudo-*F*_(3,998)_ = 110.07, *p* = 0.001; Temp × size × time for pCO_2_: Pseudo-*F*_(24,997)_ = 4.522, *p* = 0.001; figure 3). However, haemolymph $\mathrm{HC}O_{3}^{-}$ remained relatively stable over time but showed slightly elevated levels at higher temperatures (Temp effect on haemolymph $\mathrm{HC}O_{3}^{-}$: Pseudo-*F*_(3,998)_ = 15.842, *p* = 0.001; Temp × size × time: Pseudo-*F*_(24,997)_ = 1.714, *p* = 0.023; figure 3).

During recovery, i.e. on return to the respective control or OA conditions, there was no significant OA effect on any of the acid–base parameters, when considered interactions with size and time (haemolymph pH_NBS_: OA × size × time: Pseudo-*F*_(2,999)_ = 1.326, *p* = 0.251; haemolymph $\mathrm{HC}O_{3}^{-}$: OA × size × time: Pseudo-*F*_(2,999)_ = 0.364, *p* = 0.711; haemolymph pCO_2_: OA × size × time: Pseudo-*F*_(2,999)_ = 0.450, *p* = 0.635). All haemolymph acid–base parameters returned to the same level as they started prior to emersion exposure. In contrast, air temperature had a significant and interacting effect with size and time in recovery, on haemolymph pH_NBS_ (Temp × size × time: Pseudo-*F*_(6,999)_ = 2.517, *p* = 0.024; figure 3) and pCO_2_ (Temp × size × time: Pseudo-*F*_(6,999)_ = 8.416, *p* = 0.001; figure 3) but no significant effect on $\mathrm{HC}O_{3}^{-}$ (Temp × size × time: Pseudo-*F*_(6,999)_ = 2.025, *p* = 0.068; figure 3).

## 4. Discussion

The combined field and laboratory data collected in this study reveals that periods of emersion have greater physiological impact on the acid–base balance of the intertidal mussel *Mytilus edulis* than submersion in high CO_2_/low pH seawater conditions. This is true both for conditions observed in the field currently (measured *in situ*) and for predicted OA scenarios used in laboratory experiments. Mussel haemolymph *p*CO_2_ and pH varied significantly over a tidal (emersion–immersion) cycle for both of the field populations studied *in situ*, with mussels showing a significant acidosis response to periods of emersion throughout the year due to a build-up of haemolymph pCO_2_ with time during aerial exposure. This *in situ* field-collected data supports previous laboratory-based aerial exposures in bivalves reporting acidosis of haemolymph following simulated emersion (e.g. [7,36]). This acidosis response was greater in mussels from the estuarine SC population, where haemolymph *p*CO_2_ typically rose more than 4-fold during emersion (not including April data), driving a corresponding drop in pH_NBS_ from 7.71 to 6.87 over one tidal cycle. Haemolymph $\mathrm{HC}O_{3}^{-}$ levels showed very little change over tidal cycles for both populations, with the exception of a notable peak observed during the April sampling point (discussed further below). Seawater pH/pCO_2_ levels showed some variability, with a maximum range of 0.5 pH units over the sampling periods but with no clear tidal, daily, or seasonal signal at these locations.

*Mytilus* species mostly remain closed during aerial exposure, restricting the uptake or availability of environmental oxygen, and have been shown to reduce metabolic rate during emersion periods to 4–17% of their immersed rate [37] as they switch from aerobic to anaerobic metabolism [38]. Extracellular acidosis occurs via increases in respiratory and metabolic CO_2_ which builds up in the extracellular fluid, hydrating and then dissociating to form H^+^ ions and ultimately decreasing pH [16]. There is evidence that this metabolic suppression occurs more readily at lower oxygen levels in larger mussels than smaller mussels [27]. When re-immersed, the acid–base status of the mussels appears to quickly recover towards that of the overlying seawater carbonate chemistry. This lack of acid–base regulation during emersion or immersion support previous findings from studies on submerged mussel responses to OA suggesting that *M. edulis* are largely unable to regulate acid–base disturbances [22–24,39].

Interestingly, the *M. edulis* specimens sampled during April from the two distinct populations studied here exhibited quite different acid–base responses during emersion from individuals during the rest of the year. Firstly, haemolymph pCO_2_ levels reached during the April emersion period were almost twice as high as the other sampling points, reaching 1.43 kPa in SC mussels. Conversely to the other field sampling points, or indeed any of the laboratory scenarios (including the 28°C exposure where haemolymph pCO_2_ reached 1.65 kPa), mussels sampled in April did show elevated haemolymph $\mathrm{HC}O_{3}^{-}$ in association with this increase in pCO_2_. While we cannot currently explain this phenomenon, this greater haemolymph pCO_2_ under similar size and temperature conditions is suggestive of higher rates of metabolism. Since mussels in the South West of England are generally gravid in April this may be directly related to the metabolic burden of the presence of gametes. Or perhaps mussels forego metabolic suppression during emersion when gravid to protect gametes from the effects of reduced oxygen. Very little is known regarding how maturity influences tissue CO_2_ production or fundamental physiological responses in broadcast spawning invertebrates, raising interesting avenues for further investigation. Shell dissolution, leading to increases in extracellular Ca^+^ and $CO_{3}^{-}$, under this higher build-up of haemolymph pCO_2,_ may then account for the bicarbonate responses to acidosis that we observed [40,41]. It has been suggested that dissolution during emersion over tidal periods is a common feature of mytilid physiology [40,41]. A recent study demonstrated that mussels exert significant biological control over the structural integrity of their inner shell surfaces related to their energy budgets, but that significant shell erosion occurs under high seawater pCO_2_ conditions when energy is limited [40].

Laboratory exposures revealed only a small interactive effect of prior exposure to simulated OA seawater (pH_NBS_ 7.7/pCO_2_ 930 µatm) on this acidosis during emersion, with mussels previously exposed to OA conditions starting the emersion period with a slight acidosis of the haemolymph compared to those from seawater at pH_NBS_ 8.1 (haemolymph pH_NBS_ was 0.1 unit lower in large OA mussels than large control mussels). Conditions experienced during emersion then quickly overrode this prior OA exposure effect. A previous study of the Sydney rock oyster *Saccostrea glomerata* similarly revealed no effect of prior exposure to OA on haemolymph pH during emersion but did report an increase in haemolymph pCO_2_ [7], which we did not observe. We did find that haemolymph pH only recovered back to the pre-exposure levels however, i.e. for the large mussels, under OA conditions this was lower, on average, than for present-day conditions. Hence, while mussels experienced greater acidosis overall during emersion, future OA may prevent the larger mussels from recovering from this acidosis to the same degree that mussels are presently able to. Chronic OA exposures during immersion may also exacerbate these effects further. This may have implications for the future size distributions of mussels, potentially influencing the upper limit of larger mussels under future climate scenarios [7].

We found that air temperature was the primary influence on acid–base balance during emersion periods, exceeding any impact of prior OA exposure. In our laboratory experiments mussels exposed to higher temperatures of 20°C and 28°C during emersion experienced a significantly higher build-up of haemolymph pCO_2_ than those exposed to lower temperatures for the same period, driving a corresponding decrease in haemolymph pH. This is in line with the early work by Jokumsen & Fyhn [36] who demonstrated a greater degree of acidosis during emersion at 20°C than 12°C in two bivalve species. In our experiments, mussel haemolymph pH reached values as low as pH 6.74 after exposure to air temperatures of 28°C, with a corresponding haemolymph pCO_2_ maximum of 1.65 kPa. These temperatures are currently experienced *in situ* during the summer for both study populations, and induced a greater acidosis in mussels than observed for any near-future OA studies. For example, our previous work recorded haemolymph pH_NBS_ of 7.09 after exposing the same SC population of *M. edulis* to seawater pH_NBS_ values of 7.11, i.e. beyond near-future OA predicted values, for 14 days [24]. Metabolic rate is heavily influenced by temperature, as demonstrated in *M. edulis* where a 5-fold increase in metabolic rate was recorded for an increase in body temperature from 11°C to 18°C [42]. While body temperature in intertidal species during emersion is known to be driven by multiple environmental factors including microhabitat structure in ways not reflected in our laboratory exposure, hence are often quite different from the air temperature [14,26], intertidal mussels are likely already experiencing acid–base disturbances greater than those predicted for 100 years' time under OA conditions when exposed to high summer air temperatures during low tide.

We also found that larger mussels showed a greater degree of acidosis (i.e. a lower haemolymph pH) than smaller mussels during both the field observations and laboratory manipulations, irrespective of where they were collected from. To differentiate between location-driven environmental factors (such as food availability) and population differences, mussels from SC were transposed to PG during the July sampling. Again those from SC experienced a greater extracellular acidosis during emersion (but had similar acid–base physiology during immersion). The substantial difference in shell length (approx. 26 mm) between the two populations suggests size is likely to contribute to the physiological response triggered by emersion. We then compared the acid–base response of small (37–50 mm) and large (60–79 mm) mussels from the same population (SC) to a 6 h emersion period in the laboratory which further confirmed this size effect on acidosis. Previous work has demonstrated that smaller mussels tend to warm more rapidly during emersion, reaching higher body temperatures under the same conditions than larger mussels [15], however the greater metabolic CO_2_ produced by a larger body mass apparently overrides this effect here. Hence, physiological stress during emersion is greater in larger mussels compared to smaller individuals, potentially suggesting a physiological advantage to being smaller higher up the shore. Of course, numerous other factors will influence size, growth, and energetics of intertidal mussels, including feeding, predation, and wave action, hence size distributions across a shore are likely to represent complex trade-offs.

Together, these *in situ* field data and experimental laboratory data demonstrate some of the complex interactions between environmental parameters and mussel morphology that drive the acid–base physiology of an intertidal mussel, *M. edulis*. Species distribution patterns are often closely linked with environmental temperature and intertidal organisms often live very close to their thermal tolerance limits [43]. Our *in situ* field data adds to recent evidence suggesting that acid–base physiology might also play a key role in influencing these spatial patterns for intertidal species [7] and the role that near-future climate change may have on these patterns. Understanding the interaction between seawater carbonate chemistry and aerial temperature during immersion–emersion cycles, and the variability in these parameters over tidal, daily, and seasonal timescales is key to developing better predictions of the impact of future climate change on these ecologically important marine biota and where physiological tipping points may occur.

## Supplementary Material

Proc Roy Soc B Supporting Info Mangan et al Acid-Base over tidal cycles paper

## Supplementary Material

Supplementary meta-data

## Data accessibility

Additional data available in the Dryad Digital Respoitory: https://doi.org/10.5061/dryad.k11r5b9 [44].
