# Supplementary material for: Acid–base physiology over tidal periods in the mussel Mytilus edulis: size and temperature are more influential than seawater pH
Source: Proc Biol Sci. 2019 Feb 20;286(1897):20182863. doi: 10.1098/rspb.2018.2863 (PMC6408882; doi:10.1098/rspb.2018.2863)
Supplement: Proc Roy Soc B Supporting Info Mangan et al Acid-Base over tidal cycles paper [file rspb20182863supp1.docx]

**Supplementary Information: Acid-base physiology over tidal periods in the mussel *Mytilus edulis*: size and temperature are more influential than seawater pH.**

**Supplementary Methods:**

*Statistical analysis*

*Quality control*: Haemolymph data were quality checked whereby if a triplicate (or quadruplicate) had a standard deviation greater than the overall dataset standard deviation, the individual data point that was outside the standard deviation was removed from further analysis. For Starcross haemolymph pH 6 of 300 data points were removed, for Starcross haemolymph TCO_2_ 26 of 300 data points were removed. For Port Gaverne haemolymph pH 5 of 225 data points were removed, for Port Gaverne haemolymph TCO_2_ 14 of 225 data points were removed. These individual points were also removed from corresponding haemolymph pCO_2_ and HCO_3_^-^, as these were calculated from one or both of the measured pH and TCO_2_ parameters.

*Transposed mussels:* Haemolymph acid-base measurements made in the July sampling event at Port Gaverne (PG). Starcross (SC) mussels were transposed to PG to compare whether the difference in response was due to populations or local environment. Tables S1 to S3 provide the statistical analysis of differences between populations and tide height.

*Laboratory experiment – acid-base response to ‘Ocean acidification’ and emersion-recovery:* Haemolymph acid-base response during emersion were tested for effects of prior exposure to different pH conditions (control or lowered pH termed “OA”), emersion time (0 to 6 h), air temperature (7, 14, 20 and 28 °C) and mussel size (large or small). Tests were also made of the recovery period when mussels re-immersed back in the respective seawater conditions. Results of the Permanova analysis are provided in Tables S4 to S9.

**Supplementary Results:**

*Laboratory carbonate chemistry data:*

The seawater carbonate chemistry parameters for the laboratory-based ‘Ocean Acidification’ emersion-recovery experiments are also summarised in Table S10 below.

**Table S1: General Linear Model pH vs. population (SC or PG) and tide height**

***Analysis of Variance***

| Source | DF | Adj SS | Adj MS | F-Value | P-Value |
| --- | --- | --- | --- | --- | --- |
| Population | 1 | 0.2787 | 0.278727 | 45.32 | 0.000 |
| Tideheight | 22 | 4.5921 | 0.208732 | 33.94 | 0.000 |
| Population*Tideheight | 22 | 0.3771 | 0.017139 | 2.79 | 0.000 |
| Error | 103 | 0.6335 | 0.006151 |  |  |
| Total | 148 | 5.8648 |  |  |  |

**Table S2: General Linear Model HCO3 vs. population (SC or PG) and tide height**

***Analysis of Variance***

| Source | DF | Adj SS | Adj MS | F-Value | P-Value |
| --- | --- | --- | --- | --- | --- |
| Population | 1 | 2.125 | 2.12530 | 36.52 | 0.000 |
| Tideheight | 22 | 12.726 | 0.57844 | 9.94 | 0.000 |
| Population *Tideheight | 22 | 4.489 | 0.20403 | 3.51 | 0.000 |
| Error | 100 | 5.820 | 0.05820 |  |  |
| Total | 145 | 25.024 |  |  |  |

**Table S3: General Linear Model pCO_2_ vs. population (SC or PG) and tide height**

***Analysis of Variance***

| Source | DF | Adj SS | Adj MS | F-Value | P-Value |
| --- | --- | --- | --- | --- | --- |
| Population | 1 | 0.5899 | 0.589931 | 73.43 | 0.000 |
| Tideheight | 22 | 4.1131 | 0.186957 | 23.27 | 0.000 |
| Population *Tideheight | 22 | 0.8176 | 0.037165 | 4.63 | 0.000 |
| Error | 100 | 0.8034 | 0.008034 |  |  |
| Total | 145 | 6.5967 |  |  |  |

**Table S4: PERMANOVA (Permutational MANOVA) test for haemolymph pH during the emersion experiment**

**PERMDISP** (Distance-based test for homogeneity of multivariate dispersions)

Group factor: timesizetemppH

Number of permutations: 999

***Deviations from median***

F: 0.63014 df1: 143 df2: 293

P(perm): 0.88

**PERMANOVA**

Resemblance: D1 Euclidean distance

Sums of squares type: Type III (partial)

Fixed effects sum to zero for mixed terms

Permutation method: Permutation of residuals under a reduced model

Number of permutations: 999

***Factors***

| Name | Abbreviation | Type | Levels |
| --- | --- | --- | --- |
| Time | Ti | Fixed | 9 |
| Size | Si | Fixed | 2 |
| Temperature | Te | Fixed | 4 |
| Seawater pH (‘OA’) | OA | Fixed | 2 |

***PERMANOVA table of results***

| Source | df | SS | MS | Pseudo-F | P(perm) | Unique perms |
| --- | --- | --- | --- | --- | --- | --- |
| Ti | 8 | 3.4536 | 0.43171 | 65.339 | 0.001 | 999 |
| Si | 1 | 4.3614 | 4.3614 | 660.1 | 0.001 | 994 |
| Te | 3 | 1.2822 | 0.42739 | 64.686 | 0.001 | 999 |
| OA | 1 | 5.4362E-4 | 5.4362E-4 | 8.2278E-2 | 0.786 | 995 |
| TixSi | 8 | 1.2184 | 0.15229 | 23.05 | 0.001 | 997 |
| TixTe | 24 | 0.78086 | 3.2536E-2 | 4.9244 | 0.001 | 998 |
| TixOA | 8 | 0.29817 | 3.7271E-2 | 5.6411 | 0.001 | 999 |
| SixTe | 3 | 0.53956 | 0.17985 | 27.221 | 0.001 | 997 |
| SixOA | 1 | 8.8391E-2 | 8.8391E-2 | 13.378 | 0.001 | 997 |
| TexOA | 3 | 1.9541E-2 | 6.5137E-3 | 0.98586 | 0.397 | 998 |
| TixSixTe | 24 | 0.46656 | 1.944E-2 | 2.9423 | 0.001 | 997 |
| TixSixOA | 8 | 0.14182 | 1.7728E-2 | 2.6831 | 0.009 | 999 |
| TixTexOA | 24 | 0.66959 | 2.79E-2 | 4.2226 | 0.001 | 998 |
| SixTexOA | 3 | 0.24418 | 8.1394E-2 | 12.319 | 0.001 | 995 |
| TixSixTexOA | 24 | 0.54269 | 2.2612E-2 | 3.4224 | 0.001 | 998 |
| Res | 293 | 1.9359 | 6.6071E-3 |  |  |  |
| Total | 436 | 16.007 |  |  |  |  |

**Table S5: PERMANOVA (Permutational MANOVA) test for haemolymph pCO_2_ during the emersion experiment**

**PERMDISP** (Distance-based test for homogeneity of multivariate dispersions)

Group factor: timesizetemppH

Number of permutations: 999

***Deviations from median***

F: 0.95319 df1: 143 df2: 293

P(perm): 0.24

**PERMANOVA**

Resemblance: D1 Euclidean distance

Sums of squares type: Type III (partial)

Fixed effects sum to zero for mixed terms

Permutation method: Permutation of residuals under a reduced model

Number of permutations: 999

***Factors***

| Name | Abbreviation | Type | Levels |
| --- | --- | --- | --- |
| Time | Ti | Fixed | 9 |
| Size | Si | Fixed | 2 |
| Temperature | Te | Fixed | 4 |
| Seawater pH (‘OA’) | OA | Fixed | 2 |

***PERMANOVA table of results***

| Source | df | SS | MS | Pseudo-F | P(perm) | Unique perms |
| --- | --- | --- | --- | --- | --- | --- |
| Ti | 8 | 4.7753 | 0.59691 | 34.716 | 0.001 | 998 |
| Si | 1 | 5.8354 | 5.8354 | 339.38 | 0.001 | 998 |
| Te | 3 | 5.6779 | 1.8926 | 110.07 | 0.001 | 998 |
| OA | 1 | 2.0321E-2 | 2.0321E-2 | 1.1819 | 0.283 | 999 |
| TixSi | 8 | 2.0972 | 0.26216 | 15.247 | 0.001 | 998 |
| TixTe | 24 | 2.1173 | 8.8219E-2 | 5.1307 | 0.001 | 997 |
| TixOA | 8 | 0.17284 | 2.1605E-2 | 1.2565 | 0.26 | 998 |
| SixTe | 3 | 2.2269 | 0.7423 | 43.172 | 0.001 | 997 |
| SixOA | 1 | 0.10845 | 0.10845 | 6.3074 | 0.009 | 994 |
| TexOA | 3 | 0.18774 | 6.258E-2 | 3.6396 | 0.017 | 998 |
| TixSixTe | 24 | 1.8661 | 7.7756E-2 | 4.5222 | 0.001 | 997 |
| TixSixOA | 8 | 0.21471 | 2.6838E-2 | 1.5609 | 0.158 | 999 |
| TixTexOA | 24 | 0.83509 | 3.4795E-2 | 2.0237 | 0.004 | 998 |
| SixTexOA | 3 | 0.21581 | 7.1935E-2 | 4.1837 | 0.008 | 999 |
| TixSixTexOA | 24 | 1.0259 | 4.2747E-2 | 2.4861 | 0.001 | 996 |
| Res | 293 | 5.0379 | 1.7194E-2 |  |  |  |
| Total | 436 | 32.191 |  |  |  |  |

**Table S6: PERMANOVA (Permutational MANOVA) test for haemolymph HCO_3_^-^ during the emersion experiment**

**PERMDISP** (Distance-based test for homogeneity of multivariate dispersions)

Group factor: timesizetemppH

Number of permutations: 999

***Deviations from median***

F: 0.62762 df1: 143 df2: 293

P(perm): 0.891

**PERMANOVA**

Resemblance: D1 Euclidean distance

Sums of squares type: Type III (partial)

Fixed effects sum to zero for mixed terms

Permutation method: Permutation of residuals under a reduced model

Number of permutations: 999

***Factors***

| Name | Abbreviation | Type | Levels |
| --- | --- | --- | --- |
| Time | Ti | Fixed | 9 |
| Size | Si | Fixed | 2 |
| Temperature | Te | Fixed | 4 |
| Seawater pH (‘OA’) | OA | Fixed | 2 |

***PERMANOVA table of results***

| Source | df | SS | MS | Pseudo-F | P(perm) | Unique perms |
| --- | --- | --- | --- | --- | --- | --- |
| Ti | 8 | 6.4758 | 0.80947 | 7.3025 | 0.001 | 999 |
| Si | 1 | 0.67345 | 0.67345 | 6.0754 | 0.019 | 997 |
| Te | 3 | 5.2682 | 1.7561 | 15.842 | 0.001 | 998 |
| OA | 1 | 6.653 | 6.653 | 60.019 | 0.001 | 995 |
| TixSi | 8 | 1.4531 | 0.18164 | 1.6386 | 0.114 | 998 |
| TixTe | 24 | 4.4343 | 0.18476 | 1.6668 | 0.033 | 998 |
| TixOA | 8 | 3.6249 | 0.45311 | 4.0876 | 0.001 | 998 |
| SixTe | 3 | 0.29768 | 9.9227E-2 | 0.89515 | 0.451 | 999 |
| SixOA | 1 | 8.8678E-2 | 8.8678E-2 | 0.79999 | 0.35 | 998 |
| TexOA | 3 | 3.9799 | 1.3266 | 11.968 | 0.001 | 998 |
| TixSixTe | 24 | 4.5601 | 0.19001 | 1.7141 | 0.023 | 997 |
| TixSixOA | 8 | 0.41255 | 5.1569E-2 | 0.46521 | 0.888 | 999 |
| TixTexOA | 24 | 3.8625 | 0.16094 | 1.4519 | 0.089 | 997 |
| SixTexOA | 3 | 1.2081 | 0.40271 | 3.633 | 0.015 | 998 |
| TixSixTexOA | 24 | 5.0987 | 0.21245 | 1.9165 | 0.014 | 999 |
| Res | 293 | 32.479 | 0.11085 |  |  |  |
| Total | 436 | 80.637 |  |  |  |  |

**Table S7: PERMANOVA (Permutational MANOVA) test for haemolymph pH during the recovery experiment**

**PERMDISP** (Distance-based test for homogeneity of multivariate dispersions)

Group factor: timesizetemppH

Number of permutations: 999

***Deviations from median***

F: 1.2275 df1: 47 df2: 142

P(perm): 0.207

**PERMANOVA**

Resemblance: D1 Euclidean distance

Sums of squares type: Type III (partial)

Fixed effects sum to zero for mixed terms

Permutation method: Permutation of residuals under a reduced model

Number of permutations: 999

***Factors***

| Name | Abbreviation | Type | Levels |
| --- | --- | --- | --- |
| Time | Ti | Fixed | 3 |
| Size | Si | Fixed | 2 |
| Temperature | Te | Fixed | 4 |
| Seawater pH (‘OA’) | OA | Fixed | 2 |

***PERMANOVA table of results***

| Source | df | SS | MS | Pseudo-F | P(perm) | Unique perms |
| --- | --- | --- | --- | --- | --- | --- |
| Ti | 2 | 2.9698 | 1.4849 | 508.04 | 0.001 | 998 |
| Si | 1 | 0.36419 | 0.36419 | 124.61 | 0.001 | 997 |
| Te | 3 | 4.1536E-2 | 1.3845E-2 | 4.7371 | 0.005 | 999 |
| OA | 1 | 1.8156E-2 | 1.8156E-2 | 6.212 | 0.01 | 997 |
| TixSi | 2 | 0.41723 | 0.20861 | 71.375 | 0.001 | 997 |
| TixTe | 6 | 0.23594 | 3.9323E-2 | 13.454 | 0.001 | 999 |
| TixOA | 2 | 0.19817 | 9.9085E-2 | 33.901 | 0.001 | 998 |
| SixTe | 3 | 8.1532E-2 | 2.7177E-2 | 9.2985 | 0.001 | 998 |
| SixOA | 1 | 1.4979E-3 | 1.4979E-3 | 0.51251 | 0.481 | 998 |
| TexOA | 3 | 8.163E-2 | 2.721E-2 | 9.3096 | 0.001 | 998 |
| TixSixTe | 6 | 4.4144E-2 | 7.3574E-3 | 2.5173 | 0.024 | 999 |
| TixSixOA | 2 | 7.7526E-3 | 3.8763E-3 | 1.3262 | 0.251 | 999 |
| TixTexOA | 6 | 7.0166E-2 | 1.1694E-2 | 4.0011 | 0.001 | 999 |
| SixTexOA | 3 | 1.0122E-2 | 3.3741E-3 | 1.1544 | 0.324 | 998 |
| TixSixTexOA | 6 | 0.10294 | 1.7157E-2 | 5.8702 | 0.001 | 998 |
| Res | 142 | 0.41503 | 2.9228E-3 |  |  |  |
| Total | 189 | 5.055 |  |  |  |  |

**Table S8: PERMANOVA (Permutational MANOVA) test for haemolymph pCO_2_ during the recovery experiment**

**PERMDISP** (Distance-based test for homogeneity of multivariate dispersions)

Group factor: timesizetemppH

Number of permutations: 999

***Deviations from median***

F: 1.8507 df1: 47 df2: 142

P(perm): 0.029

**PERMANOVA**

Resemblance: D1 Euclidean distance

Sums of squares type: Type III (partial)

Fixed effects sum to zero for mixed terms

Permutation method: Permutation of residuals under a reduced model

Number of permutations: 999

***Factors***

| Name | Abbreviation | Type | Levels |
| --- | --- | --- | --- |
| Time | Ti | Fixed | 3 |
| Size | Si | Fixed | 2 |
| Temperature | Te | Fixed | 4 |
| Seawater pH (‘OA’) | OA | Fixed | 2 |

***PERMANOVA table of results***

| Source | df | SS | MS | Pseudo-F | P(perm) | Unique perms |
| --- | --- | --- | --- | --- | --- | --- |
| Ti | 2 | 4.5581 | 2.2791 | 652.63 | 0.001 | 999 |
| Si | 1 | 0.50212 | 0.50212 | 143.79 | 0.001 | 994 |
| Te | 3 | 1.3763 | 0.45878 | 131.37 | 0.001 | 999 |
| OA | 1 | 6.6083E-2 | 6.6083E-2 | 18.923 | 0.002 | 997 |
| TixSi | 2 | 0.57143 | 0.28572 | 81.817 | 0.001 | 997 |
| TixTe | 6 | 1.27 | 0.21167 | 60.613 | 0.001 | 998 |
| TixOA | 2 | 1.8487E-2 | 9.2433E-3 | 2.6469 | 0.067 | 999 |
| SixTe | 3 | 0.17825 | 5.9417E-2 | 17.015 | 0.001 | 999 |
| SixOA | 1 | 5.4146E-3 | 5.4146E-3 | 1.5505 | 0.215 | 998 |
| TexOA | 3 | 2.4995E-2 | 8.3318E-3 | 2.3859 | 0.078 | 999 |
| TixSixTe | 6 | 0.17633 | 2.9389E-2 | 8.4157 | 0.001 | 999 |
| TixSixOA | 2 | 3.1444E-3 | 1.5722E-3 | 0.45022 | 0.635 | 999 |
| TixTexOA | 6 | 9.3703E-2 | 1.5617E-2 | 4.4721 | 0.001 | 999 |
| SixTexOA | 3 | 2.5572E-2 | 8.5239E-3 | 2.4409 | 0.07 | 999 |
| TixSixTexOA | 6 | 8.4922E-2 | 1.4154E-2 | 4.053 | 0.001 | 999 |
| Res | 142 | 0.49588 | 3.4921E-3 |  |  |  |
| Total | 189 | 9.4823 |  |  |  |  |

**Table S9: PERMANOVA (Permutational MANOVA) test for haemolymph HCO_3_^-^ during the recovery experiment**

**PERMDISP** (Distance-based test for homogeneity of multivariate dispersions)

Group factor: timesizetemppH

Number of permutations: 999

***Deviations from median***

F: 1.5024 df1: 47 df2: 142

P(perm): 0.073

**PERMANOVA**

Resemblance: D1 Euclidean distance

Sums of squares type: Type III (partial)

Fixed effects sum to zero for mixed terms

Permutation method: Permutation of residuals under a reduced model

Number of permutations: 999

***Factors***

| Name | Abbreviation | Type | Levels |
| --- | --- | --- | --- |
| Time | Ti | Fixed | 3 |
| Size | Si | Fixed | 2 |
| Temperature | Te | Fixed | 4 |
| Seawater pH (‘OA’) | OA | Fixed | 2 |

***PERMANOVA table of results***

| Source | df | SS | MS | Pseudo-F | P(perm) | Unique perms |
| --- | --- | --- | --- | --- | --- | --- |
| Ti | 2 | 33.856 | 16.928 | 198.44 | 0.001 | 999 |
| Si | 1 | 0.35559 | 0.35559 | 4.1685 | 0.035 | 996 |
| Te | 3 | 34.689 | 11.563 | 135.55 | 0.001 | 999 |
| OA | 1 | 1.5091 | 1.5091 | 17.691 | 0.001 | 996 |
| TixSi | 2 | 0.41792 | 0.20896 | 2.4496 | 0.065 | 997 |
| TixTe | 6 | 7.9774 | 1.3296 | 15.586 | 0.001 | 999 |
| TixOA | 2 | 3.1102 | 1.5551 | 18.23 | 0.001 | 998 |
| SixTe | 3 | 0.14621 | 4.8735E-2 | 0.57131 | 0.622 | 999 |
| SixOA | 1 | 0.20648 | 0.20648 | 2.4206 | 0.123 | 999 |
| TexOA | 3 | 2.3251 | 0.77504 | 9.0856 | 0.001 | 999 |
| TixSixTe | 6 | 1.0365 | 0.17275 | 2.0251 | 0.068 | 999 |
| TixSixOA | 2 | 6.2055E-2 | 3.1028E-2 | 0.36373 | 0.711 | 999 |
| TixTexOA | 6 | 4.56 | 0.75999 | 8.9092 | 0.001 | 998 |
| SixTexOA | 3 | 0.35533 | 0.11844 | 1.3885 | 0.249 | 999 |
| TixSixTexOA | 6 | 0.49236 | 8.206E-2 | 0.96197 | 0.45 | 999 |
| Res | 142 | 12.113 | 8.5304E-2 |  |  |  |
| Total | 189 | 103.83 |  |  |  |  |

**Table S10: Measured seawater conditions for the two pH treatments, pH 8.10 (control) and pH 7.70 (‘OA’), showing mean ± SD, for measured seawater temperature, salinity, pH and dissolved inorganic carbon (DIC), and calculated total alkalinity (TA), pCO2, bicarbonate (HCO_3_^-^) and carbonate (CO_3_^2-^) ion concentrations. These latter parameters were calculated from measured temperature, salinity, pH and DIC, using CO2sys (1) using K1 and K2 values refitted by Dickson and Millero (2) from Mehrbach et al. (3), and KSO4 determined by Dickson (4)**

| Experiment | Treatment | Temperature (°C) | Salinity | pH_NBS_ | DIC  (µmol kg^-^¹) | TA  (µmol kg^-^¹) | pCO₂ (µatm) | HCO₃^-^  (µmol kg^-^¹) | CO₃²^-^  (µmol kg^-^¹) |
| --- | --- | --- | --- | --- | --- | --- | --- | --- | --- |
| Pre-Emersion | pH 8.10 | 13.2 ± 0.0 | 30.6 ± 0.0 | 8.11 ± 0.00 | 2077 ± 44 | 2287 ± 47 | 342 ± 8 | 1907 ± 40 | 156 ± 3 |
| Pre-Emersion | pH 7.70 | 13.2 ± 0.0 | 30.6 ± 0.0 | 7.70 ± 0.00 | 2145 ± 82 | 2204 ± 84 | 927 ± 30 | 2042 ± 78 | 66 ± 3 |
| Recovery | pH 8.10 | 13.2 ± 0.0 | 30.6 ± 0.0 | 8.11 ± 0.00 | 2084 ± 67 | 2297 ± 72 | 341 ± 11 | 1914 ± 62 | 157 ± 5 |
| Recovery | pH 7.70 | 13.2 ± 0.0 | 30.6 ± 0.0 | 7.70 ± 0.00 | 2144 ± 83 | 2201 ± 84 | 942 ± 32 | 2041 ± 79 | 65 ± 3 |

**Figure S1**


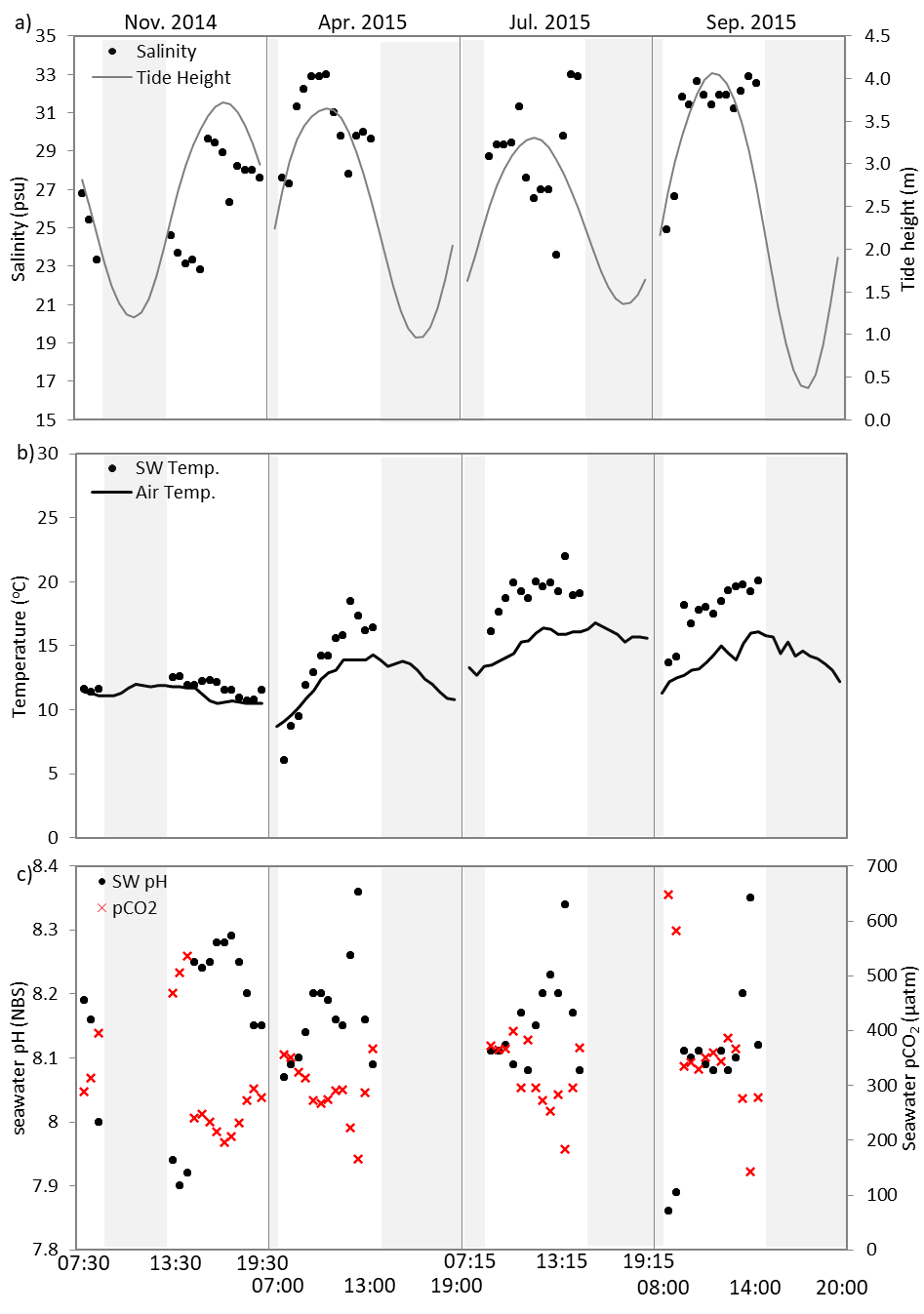


**Figure S1:** Environmental conditions for Starcross during the sampled tidal cycles in November 2014, April 2015, July 2015 and September 2015 showing: a) Salinity and tidal height (m); b) seawater temperature and air temperature (ºCº); and c) seawater pH_NBS_ and seawater pCO_2_ (µatm). Shaded sections represent periods of emersion.

**Figure S2**


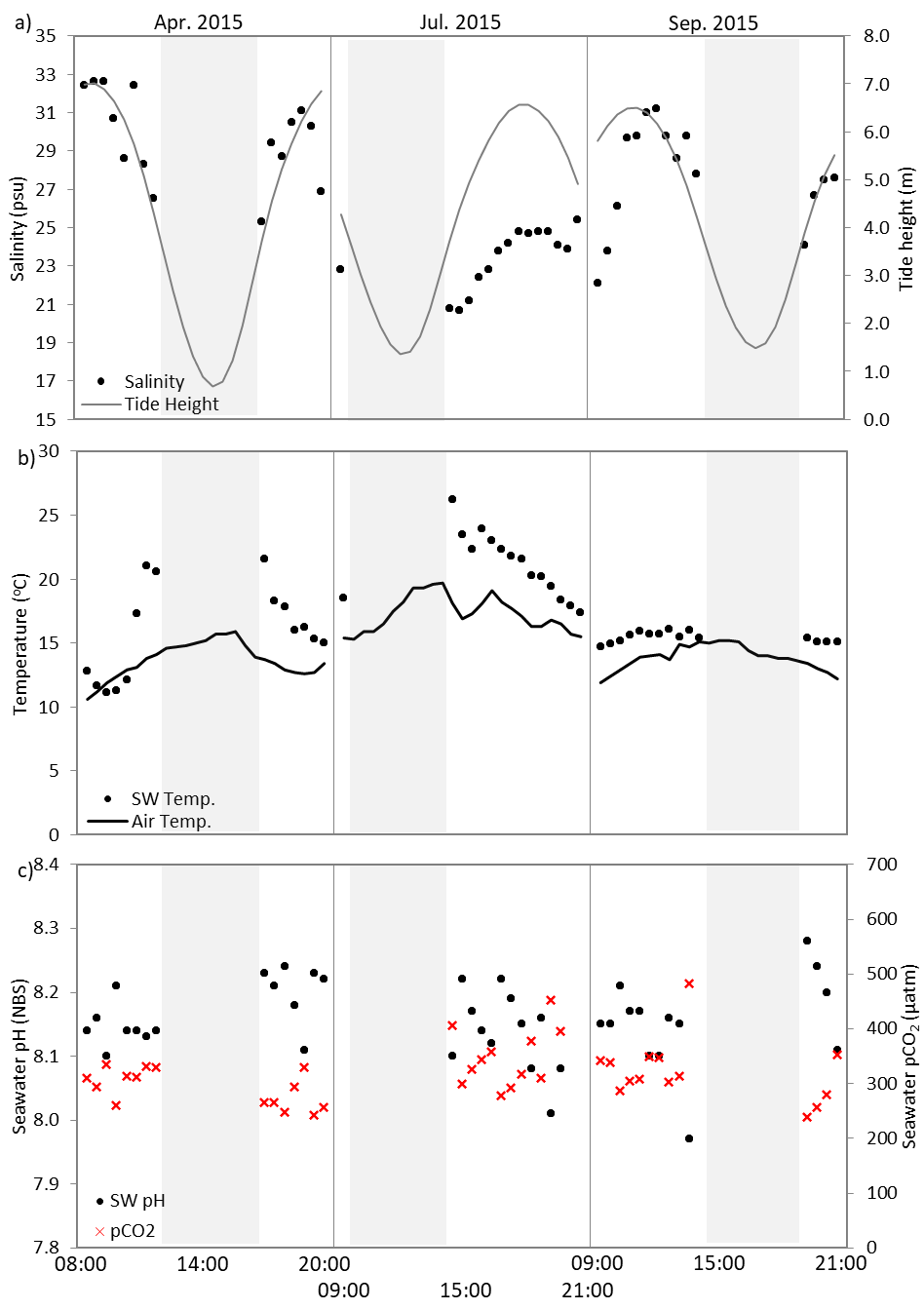


**Figure S2:** Environmental conditions for Port Gaverne during the sampled tidal cycles in November 2014, April 2015, July 2015 and September 2015 showing: a) Salinity and tidal height (m); b) seawater temperature and air temperature (°C); and c) seawater pH_NBS_ and seawater pCO_2_ (µatm). Shaded sections represent periods of emersion.

Additional data table S1 (separate excel file)

All the data generated from this study is provided in an additional excel file entitled “Mangan et al Supplementary meta-data.xls”, which contains a metadata spreadsheet “Metadata”, a spreadsheet containing field data “FieldData”, a spreadsheet containing the acid-base data from the laboratory study “LabDataBiol”, and a spreadsheet containing the carbonate chemistry data from the laboratory study “LabDataChem”.

**References**

1. Pierrot D., Lewis E., Wallace D.W.R. 2006 MS Excel program developed for CO_2_ system calculations.
2. Dickson A.G., Millero F.J. 1987 A comparison of the equilibrium-constants for the dissociation of carbonic-acid in seawater media. *Deep-Sea Research Part a-Oceanographic Research Papers* **34**(10), 1733-1743. (doi:10.1016/0198-0149(87)90021-5).
3. Mehrbach C., Culberso.Ch, Hawley J.E., Pytkowic.Rm. 1973 Measurement of apparent dissociation-constants of carbonic-acid in seawater at atmospheric-pressure. *Limnology and Oceanography* **18**(6), 897-907.
4. Dickson A.G. 1990 Standard potential of the reaction - AgCl(s)+1/2H_2_(g)=Ag(s)+HCl(aq) and the standard acidity constant of the ion HSO_4_^-^ in synthetic sea-water from 273.15-k to 318.15-k. *Journal of Chemical Thermodynamics* **22**(2), 113-127. (doi:10.1016/0021-9614(90)90074-z).
